# Radar based technology for non-contact monitoring of accumulation of blood in the head: A numerical study

**DOI:** 10.1371/journal.pone.0186381

**Published:** 2017-10-12

**Authors:** Moshe Oziel, Rafi Korenstein, Boris Rubinsky

**Affiliations:** 1 Department of Physiology and Pharmacology, Sackler School of Medicine, Tel-Aviv University, Tel-Aviv, Israel; 2 Department of Mechanical Engineering, University of California at Berkeley, Berkeley, CA, United States of America; Beijing University of Posts and Telecommunications, CHINA

## Abstract

**Background:**

This theoretical study examines the use of radar to continuously monitor “accumulation of blood in the head” (ACBH) non-invasively and from a distance, after the location of a hematoma or hemorrhage in the brain was initially identified with conventional medical imaging. Current clinical practice is to monitor ABCH with multiple, subsequent, conventional medical imaging. The radar technology introduced in this study could provide a lower cost and safe alternative to multiple conventional medical imaging monitoring for ACBH.

**Materials and methods:**

The goal of this study is to evaluate the feasibility of using radar to monitor changes in blood volume in the brain through a numerical simulation of ACBH monitoring from remote, with a directional spiral slot antennae, in 3-D models of the brain. The focus of this study is on evaluating the effect of frequencies on the antennae reading. To that end we performed the calculations for frequencies of 100 MHz, 500 MHz and 1 GHz.

**Results and discussion:**

The analysis shows that the ACBH can be monitored with radar and the monitoring resolution improves with an increase in frequency, in the range studied. However, it also appears that when typical clinical dimensions of hematoma and hemorrhage are used, the variable ratio of blood volume radius and radar wavelength can bring the measurements into the Mie and Rayleigh regions of the radar cross section. In these regions there is an oscillatory change in signal with blood volume size. For some frequencies there is an increase in signal with an increase in volume while in others there is a decrease.

**Conclusions:**

While radar can be used to monitor ACBH non-invasively and from a distance, the observed Mie region dependent oscillatory relation between blood volume size and wavelength requires further investigation. Classifiers, multifrequency algorithms or ultra-wide band radar are possible solutions that should be explored in the future.

## Introduction

It is well established that “Intracerebral hemorrhage is a serious medical condition for which outcome can be impacted by early, aggressive care.” [1]. Accumulation of blood in the brain can be of two types: hemorrhage, which is leakage of blood outside the blood vessel and hematoma, which is accumulation of blood within tissue planes. This study deals with monitoring “accumulation of blood in the head” (ACBH), in general, regardless of the mechanism of accumulation. However, when relevant, we will discuss the particular modality of ACBH. Medical imaging, including ultrasound (US) computed tomography (CT) and magnetic resonance imaging (MRI) are essential for the diagnostics and treatment of intracranial hemorrhage and hematomas [2–6]. In fact, neuroimaging is considered mandatory [1]. Hematoma and hemorrhage growth correlates with neurological deterioration and increased morbidity and mortality [7–9]. Therefore, follow up of intracranial hemorrhage with imaging has become an important element in treatment of brain injuries [10] and is part of the clinical routine [1]. However, not all medical conditions benefit from medical imaging follow up. On one hand, follow up medical imaging is valuable and required [11], on the other there are circumstances in which it does not produce added value [12, 13]. Multiple medical imaging sessions increase the cost to the health care system and inconvenience the patients. Furthermore, multiple medical imaging may have substantial detrimental side effect, such as increased risk for cancer, in particular to children [14–18]. Taken as a whole, the disadvantages of repeat head imaging may surpass the advantages. However, there are cases in which continuous monitoring is required. The radar based technology of this study, may provide a solution.

The radar based technology introduced in this study, is an outgrow of a technology developed by us earlier, in which we used multifrequency phase shift spectroscopy to detect changes in fluids in the body [19–23]. Multifrequency phase shift spectroscopy employs non-ionizing, low energy multifrequency electromagnetic waves and measures the phase shift, as the waves are transmitted between two electromagnetic coils that brace the tissue that is probed. The technology has found applications to monitoring edema, hematoma and cerebrovascular reactivity in the brain [22, 23]. Recently, a clinical version of the technology also known as “Volumetric Integral Phase-shift Spectroscopy (VIPS)” was able to detect intracranial fluid shifts during hemodialysis [24]. The VIPS technology employs two coils that are positioned across the head, in such a way as to examine the entire brain. Recent versions of the device employ rigid connection to the skull.

The goal of this paper is to introduce and evaluate the feasibility of a new diagnostic technology that can monitor the growth of an ACBH in the brain when the location of the ACBH is known from previous conventional medical imaging, as an alternative to continuous monitoring with conventional methods of medical imaging. Unlike the multifrequency phase shift spectroscopy method, the technology introduced here uses only one spiral antenna, with a reflector, and employs the principles of radar. This facilitates convenient and unrestricted placement of the antenna anywhere around the head, at a location that is optimized for the particular ACBH that is monitored. The electromagnetic energy dose is low and the electromagnetic waves are non-ionizing. The technology may become a convenient means to monitor the change in size of a specific ACBH, when the location of the ACBH is known from conventional medical imaging; as an alternative to repeat medical imaging with conventional medical imaging means.

Radar employs the reflection of electromagnetic waves from interfaces between surfaces with different electromagnetic permittivity. It is used to probe an object of interest, without contact, from a distance. Radar devices consist of an emitting and receiving antenna, to emit and receive the electromagnetic waves. Radar was originally developed to detect large objects at far distance for such applications as air traffic control and surveillance, detection of sea vessels and navigation. However, the unique ability of electromagnetic waves to penetrate non-metallic objects suggested other uses, such as ground penetrating radar [25] for applications such as high resolution mapping of soil [26] and in construction. For example, a frequency of 2 GHz was used to monitor water seepage from a PVC pipe hidden in a concrete wall [27]. Among other applications, our group has developed radar technology to monitor human movements, even through walls, for analysis of neurological disorders associated with motion, such as Parkinson [28, 29].

A 1972 review on the use of electromagnetic waves in the spectrum between 1 MHz and 300 GHz in medicine, is found in [30]. Johnson and Guy, [30] reference earlier 1958 work by Moskalenko [31] in which changes in microwave or shortwave reflectance and transmission, caused by significant variation in parameters such as respiratory volume changes, are assessed. Thomas McEwan, patented an implementation of (ultra-wideband) UWB radar for remote vital signs monitoring [32]. A subsequent review on the use of radar in medical measurements is found in a 2002 paper [33]. In a 2005 paper, Paulson et al, [34] summarize advances in UWB for diagnostics, to that date. An excellent 2010 review of the use of radar in medical applications is found in [35]. With regards to the use of radar in the brain, Haddad et al. describes a technology to “detect and localize” blood pooling near the surface of the body, including sub-dural and epi-dural hematomas. The technology maps the brain with a near field microwave-antenna [36]. Recently, a series of papers by Mobashsher et al, [37–42] describe the use of ultra-wide band (UWB) antenna in the microwave range for developing an image of the brain, to produce an image of a hematoma and the surrounding brain tissue.

This study explores the feasibility of using radar, as a simple means to monitor changes in the volume of an ACBH, when it’s location is known from previous imaging with conventional means. To this end we examine the use of a single spiral radar antenna to monitor the growth of an ACBH, when the location of the ACBH is known, *a-priori*. A primary goal of the study is to evaluate the effect of frequency on the measurements.

## Materials and methods

### Mathematical model

**Fig 1:**
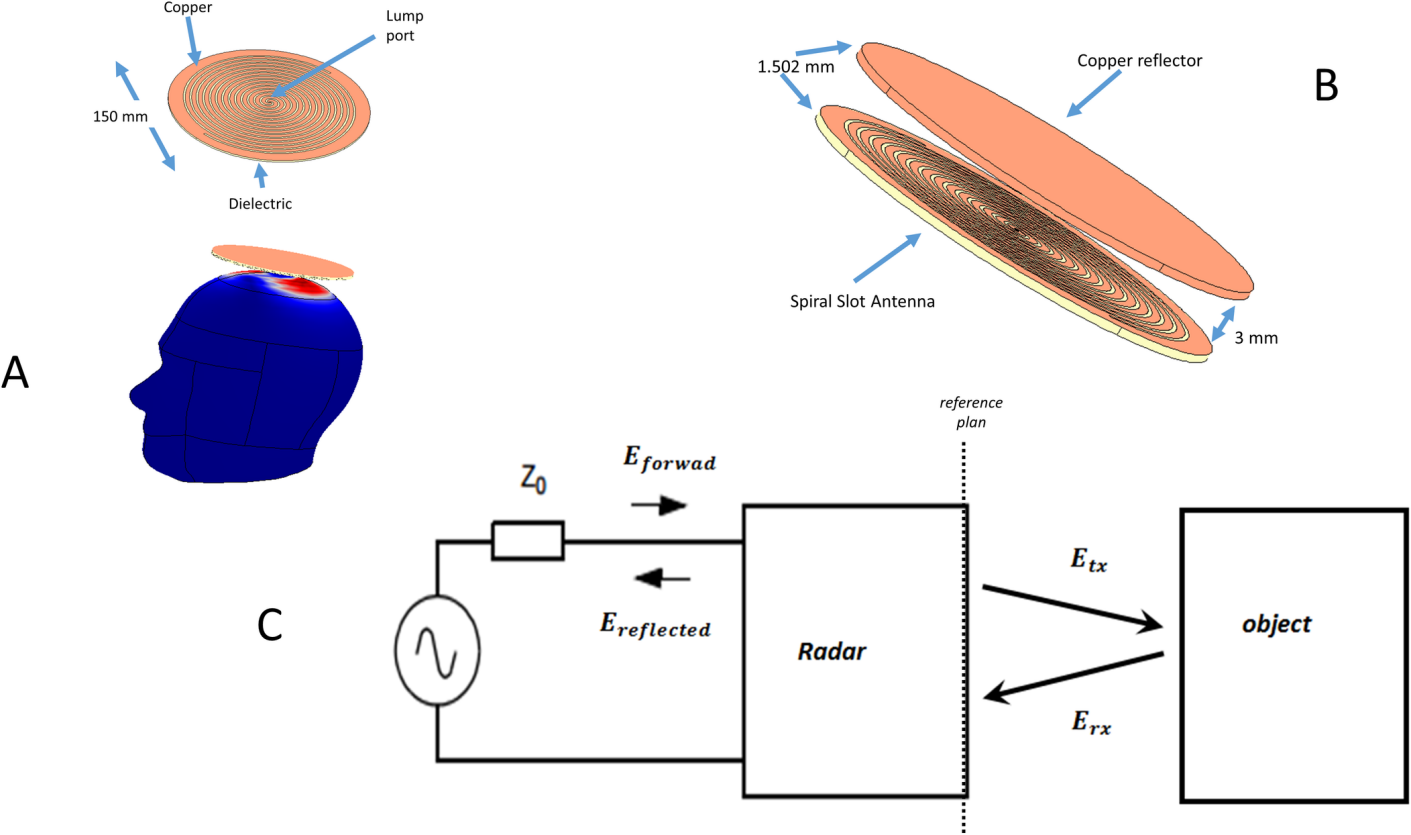
Radar system for monitoring accumulation of blood in the brain. (A) Scheme of the structure of the spiral slot copper antenna and of its placement relative to the head; (B) Magnified scheme of the antenna and the reflector. The structure of the antenna in the direction from the head outward is, as follows: i) a dielectric layer on top of which rests; ii) the spiral slot antenna, which is made of a thin film of copper in which spiral form slots are machined; iii) a gap of air; iv) a copper reflector. (C) Schematic diagram of radar operation.

Fig 1A shows a schematic of the spiral antenna and of the placement of the antenna relative to the head. [43, 44]. A cross section through the antenna is shown in Fig 1B. The structure of the antenna, in the direction from the head outward is, as follows: a) a dielectric layer on top of which rests, b) the spiral slot antenna, which is made of a thin film of copper in which the spiral form slots are machined, c) a gap of air, d) a copper reflector.

The mathematical model describing the electromagnetic phenomena in this study requires the use of the complete set of Maxwell equations; because at frequencies above about 400 MHz, the steady or quasi steady approximations fail. We have used the COMSOL RF Module Version 5.2. For high frequency models, voltage is not a well-defined variable and it is necessary to use scattering parameters (S), defined in terms of electric fields. Scattering parameters (or S-parameters) are complex, frequency dependent variables describing the transmission and reflection of electromagnetic waves at different ports of a device. S-parameters originate from transmission-line theory and are defined in terms of transmitted and reflected voltage waves.

The derivation that follows is typical to analysis of electromagnetic fields using scattering parameters [45]. The equivalent simple diagram of the radar head model is shown 1C. The diagram shows a voltage source feeding into the radar with a source impedance of *Z*_0_. If we define $a=\frac{E_{forward}}{\sqrt{Z_{0}}}$ and $b=\frac{E_{reflected}}{\sqrt{Z_{0}}}$ as the square root of the forward and reflected incident electromagnetic waves, we can define the scattering function *S*_11_ as, $S_{11}=\frac{b}{a}$ [45], and the input impedance can be express as:

$$Z_{L}=Z_{0}\left[\frac{1+S_{11}}{1-S_{11}}\right]$$ (1)

When an external electromagnetic wave impinges on the radar antenna with an amplitude of *E*_*rx*_ it will lead to a change of the *E*_*reflected*_, and consequently, will result in a change in the radar input impedance, Z_L_. In practical situation this change can be measured with a vector analyzer.

An expression derived from the Maxwell equations, for small changes in the radar input impedance, is given by [46]:

$${\Delta Z}_{L}=-\frac{1}{I^{2}}∭\left[(\delta \sigma -i\omega \delta \varepsilon )E∙E^{\left(0\right)}-(i\omega \delta \mu )H∙H^{\left(0\right)}\right]dV$$ (2)

Where E, H are the electric field and the magnetic field, respectively, in the presence of an inhomogeneity (an ACBH), *E*^(0)^, *H*^(0)^ are the electric field and the magnetic field in the absence of the inhomogeneity, *δσ*, *δε and δμ* are the changes in conductivity, permeability and permittivity.

In this study, we seek to evaluate the feasibility of using the radar to monitor the change in size of an ACBH in the head, in time and the effect of frequency on the measurement. To this end we evaluate the changes in the radar antenna input impedance, between the original value at the start of the measurements and values of the radar input impedance at a later time. In Eq (3) we mark the time when the base line reading is taken, with (*t*_1_). This is the instant at which the conventional medical image was taken. The second point in time (*t*_2_), is later; it can also be continuous in time. We can analyze the change in size of the ACBH, by analyzing the changes in readings at the antenna, between the time instances. These changes are a result of the changes in amplitude and phase of the reflection from the head to the antennae, due to the change in size of the ACBH. There are various ways to compare the readings at the different times. Here, we use the absolute value of the changes of the impedance between *t*_1_ (the base line) and *t*_2_:

$$\Delta Z=abs(Z_{L,t2}-Z_{L,t1})$$ (3)

$$abs(Z)=abs(a_{z}+{ib}_{z})=\sqrt{a_{z}^{2}+b_{z}^{2}}).$$ (4)

It is important to note that our choice to use the absolute value of the changes in transmitted impedance is just one of many possible ways to analyze the combined changes in both amplitude and phase using the definition. Many other different combinations of change in amplitude and phase with different weight for the amplitude and the phase, are possible. For instance, it is possible to use only the phase shift, as we did in [19]. In this paper we will use Eq (3), which weights equally both, amplitude and phase.

### The antennae configuration

The schematic of the antennae used in this study is shown in Fig 1A and Fig 1B. We chose to use a spiral slot antenna [43, 44] for several reasons. The spiral slot antenna was chosen for its directionality and wideband properties. The directionality of the antenna, facilitates pointing the antenna to the location of the hematoma identified by conventional medical imaging. In addition this antennae configuration has a large gain over a wide bandwidth. Earlier studies have shown that circular polarization improves the depth resolution of ultra-wide band microwave imaging radar systems, relative to linear polarization [47]. Circular polarization provides a good approximation of the shape and size of the objects [47].

The analysis assumes a spiral slot antenna [44]. The detailed schematic is shown in Fig 1B. The antenna is made of two main parts. The part farthest away from the head is a reflector made of copper, whose function is to reflect most of the energy towards the antenna and the head. The antenna is made of a copper disk with a radius of 75 mm and a thickness of 1.5 mm. The second part (closer to the head) is the spiral slot antenna. The part of the spiral slot antenna, facing the head, is a dielectric 1.5 mm thick and 75 mm in diameters. It is assumed that the dielectric plate has typical properties for its’ function[48]: *ε*_*r*_ = 3.38 (relative permittivity). *μ*_*r*_ = 1 (relative permeability) and *σ* = 0 (electrical conductivity). On top of the dielectric, facing the reflector is a two-arm Archimedean spiral slot with 14 turns patterned on a thin single-sided copper sheet. The slot follows the path of the equation *x* = 1.5 ∙ *s* ∙ cos(*s*), *y* = 1.5 ∙ *s* ∙ sin(*s*), where s, ranges from 0 to 14 *π* and the width of the slots is 1.5 mm. The two parts are along the same center line and the distance between the parts is 3 mm.

To enable comparison of the changes in impedance due to changes in ACBH size at different frequencies we calibrated the input voltage of the lumped port so that at the location (x = 0, y = 0 z = -3mm) the value of the normal to the electric field in free air is exactly 79.05 V/m, for each frequency.

### The models, mesh and boundary conditions

To isolate, mathematically, the effect of the changes in brain ACBH size on the antenna impedance, from artifacts caused by environment effects—the boundary of the analyzed domain must perfectly absorb incoming waves from all angles, without reflections. This is achieved mathematically through a perfectly matched layer (PML) at the model's boundary. PMLs are layers that absorbs all radiated waves with small reflections[49].

**Fig 2:**
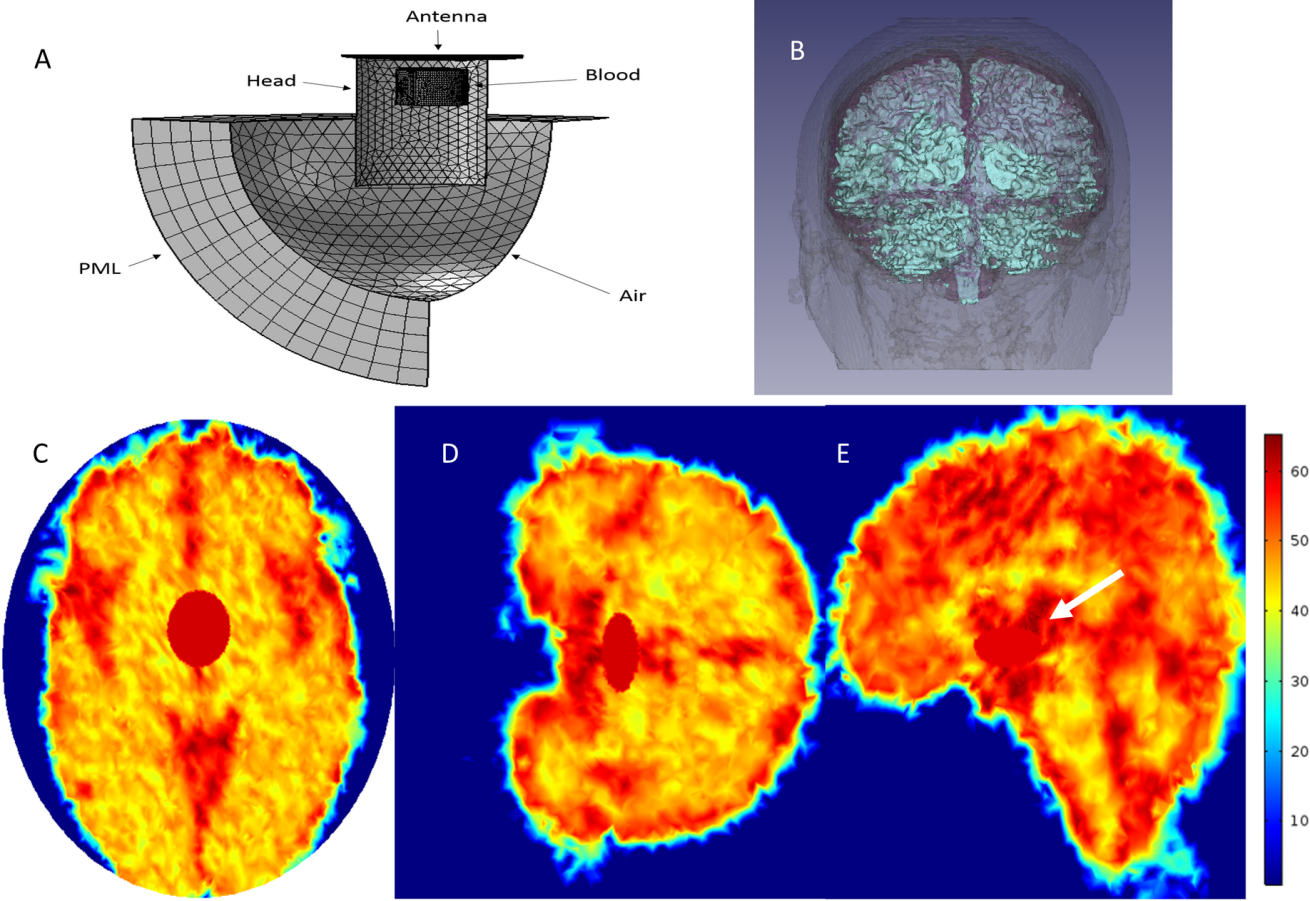
Details of the analyzed configuration: Mesh structure and head segmentation. (A) Typical mesh configuration. The analysis was performed by defined electromagnetic properties in each element of the mesh. B) The head image after segmentation. (C) (D) and (E) The permittivity maps for 1GHz on the mesh representing the brain slices (including ACBH simulation) in an axial coronal and sagittal orientation, respectively.

Fig 2A, shows a schematic of the mesh used in analyzing the effect of a ACBH on the impedance of a radar antennae. Fig 2A shows the PML in the form of a hollow sphere with a radius of 30 cm and thickness of 10 cm. The air (the internal sphere inside the PML) maximum mesh element size is 2cm. The antenna maximum mesh size is 1cm with an average value of 0.5 mm. The head, was modeled, by assigning the appropriate electromagnetic values to each element, in what is marked as the head mesh in Fig 2A. Similarly, for the ACBH. The size of the mesh elements used for the head ranged from 1cm to 1mm. The total number of mesh elements is 308,302 and the number of degrees of freedom in this simulation is 2,187,010.

In the analysis whose results are presented here, we replaced the cylindrical shape of the brain in Fig 2A with a mesh formed from a multidimensional MRI image of a real head[50], Fig 2B. The web site provides a set of 60 images from coronal scans of MRI images of a healthy, 43 years old male. The scan was made with a 3 Tesla, GE MRI. The MR’s images voxel size is 1:1:3 mm. In order to distinguish between the different brain parts, we process and segment the images using 3D slicer software[51]. For use with the 3D slicer we classified the brain elements into 4 types of tissue: white matter (WM), gray matter (GM), cerebrospinal fluid (CSF) and the skull bone. For each brain’s element we coupled the appropriate electrical properties (Permittivity and Conductivity) per frequency using ITIS’s dielectric tissue properties database[52]. We packed the spatial dielectric properties as a Matlab 3D interpolation object and used COMSOL Multiphysics software (RF Module 5.2) that allows the use of Matlab functions to describe material properties. Examples of the permittivity maps in axial coronal and sagittal slices are shown in Fig 2C, 2D and 2E, respectively.

## Results and discussion

To facilitate a better understanding of the results, we will introduce an important concept in radar analysis—the radar cross section (RCS), *σ*. A simplistic definition of the radar cross section is that the RSC is a measure of how detectable an object is with radar. A more rigorous definition is that the RCS is the ratio of backscatter power per steradian (unit solid angle) in the direction of the radar (from the target), to the power density that is intercepted by the target[53]. A target’s RCS (σ) can define as:

σ=ProjectedcrosssectionxReflectivityxDirectivity (5)

Where reflectivity is the percent of intercepted power reradiated by the target. It is important to note that the reflectivity is directly depend on the wave frequency. Directivity is the part of the power scattered back in the radar’s direction and the projected cross section is the area of the target viewed by the radar.

For a perfectly conducting sphere, the radar equation has an analytical solution. The solution has three regions, specified by the relative size of the wave length, *λ*, to the sphere radius, r. The **“**optical region” (far field) where *r* ≫ *λ* (or the characteristic dimension in case of other shapes); the Rayleigh region in which the radius of the sphere is smaller than the radius of the wavelength, *r* ≪ *λ* and the intermediary region between the optical and Rayleigh regions, called the Mie region or resonance region.

**Fig 3:**
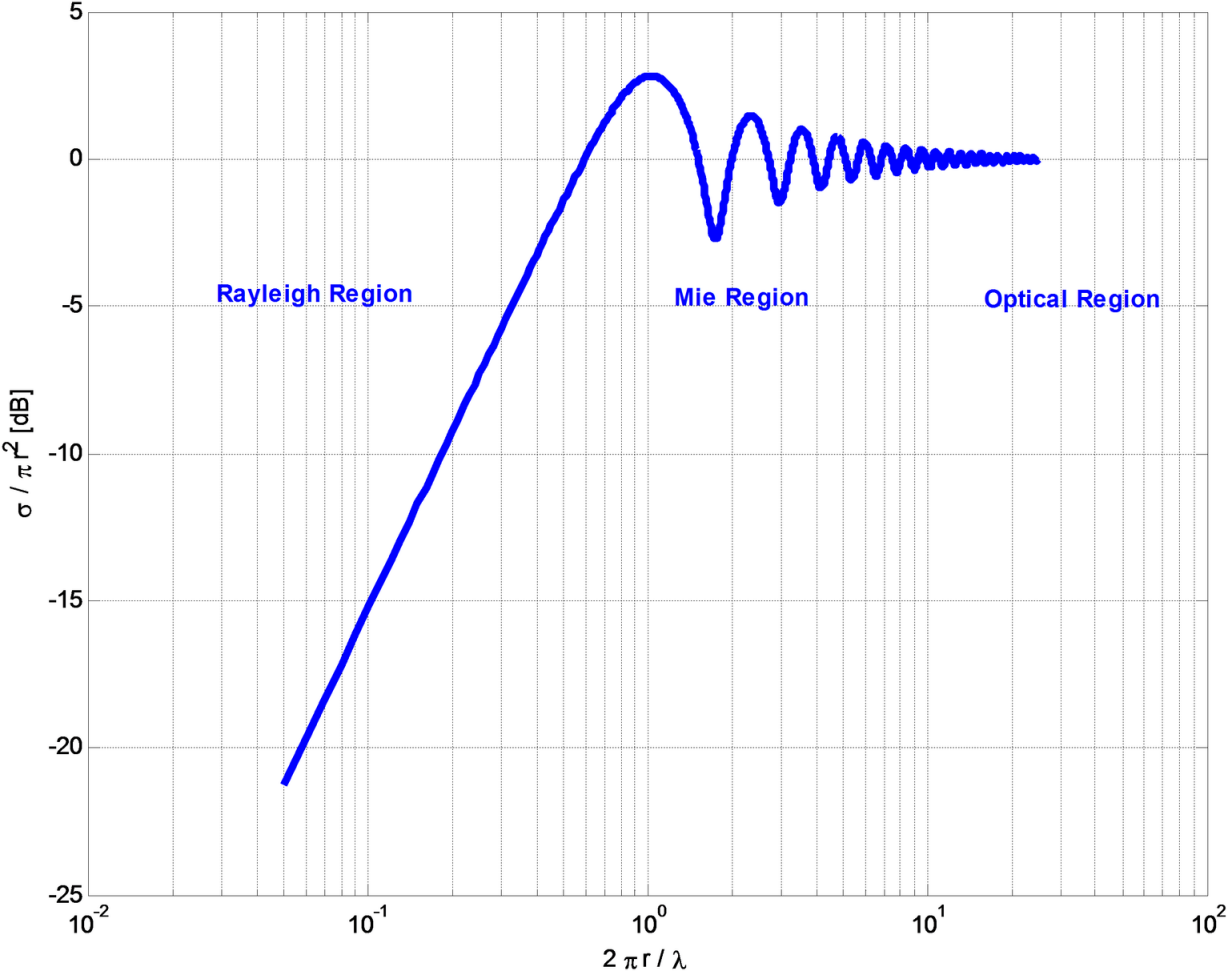
The radar cross section (RCS), *σ*, as a function of the wavelength, *λ*. The abscissa is the radius of the conducting sphere normalized with respect to the wavelength. The ordinate is the radar cross section, *σ*, normalized with respect to the conductor sphere geometrical cross section. The dependence of RCS on wavelength exhibits three distinct patterns of behavior. Of interest to this study is the Mie region in which the object geometrical dimension is on the order of magnitude of the wavelength, and dependence of RCS on frequency takes an oscillatory shape.

In the optical region, the RCS takes a maximal value and the value does not depend on the wavelength, *σ*_*max*_ = *πr*^2^ (*λ* ≪ *r*) [54]. In the Rayleigh region, *σ* ≈ 9*πr*^2^ (*kr*)^2^ where $k=\frac{2\pi }{\lambda }$ (*r* ≪ *λ*). In the Mie region (resonance), the RCS is oscillating in a form prescribed by Hankel and Bessel functions. Fig 3, is a classical diagram of the relation between RCS and wavelength[54], brought here to facilitate a discussion of the results of this study.

ACBH change in size with time was examined in three configurations. The configurations are:

1. A simulated ACBH in free air.
2. A simulation of ACBH in a computer model of a real head.
3. A simulation of ACBH growth from various clinical data in the literature.

The primary goal of this study is to evaluate the feasibility of monitoring of ACBH with radar. In the problem analyzed here, the blood volume is not constant and changes in time. The change in blood volume results in changes in RCS, which can bring the RCS through the Mie and Rayleigh regions. The precise details of the way in which the RCS changes with changes in blood volume is dependent on the radar frequency. To generate a fundamental understanding of the effect of frequency on the ability of radar to monitor ACBH, we studied each individual frequency separately. We examined three frequencies: 100 MHz, 500 MHz and 1 GHz, that were chosen because they encompass the range between frequencies in which steady and quasi-steady models apply and those that require the solution of the complete Maxwell equations. These frequencies also illustrate the effect of changes in ACBH in the Mie and Rayleigh regions of the radar cross section, on the radar readings.

### Simulated ACBH in free air

**Fig 4:**
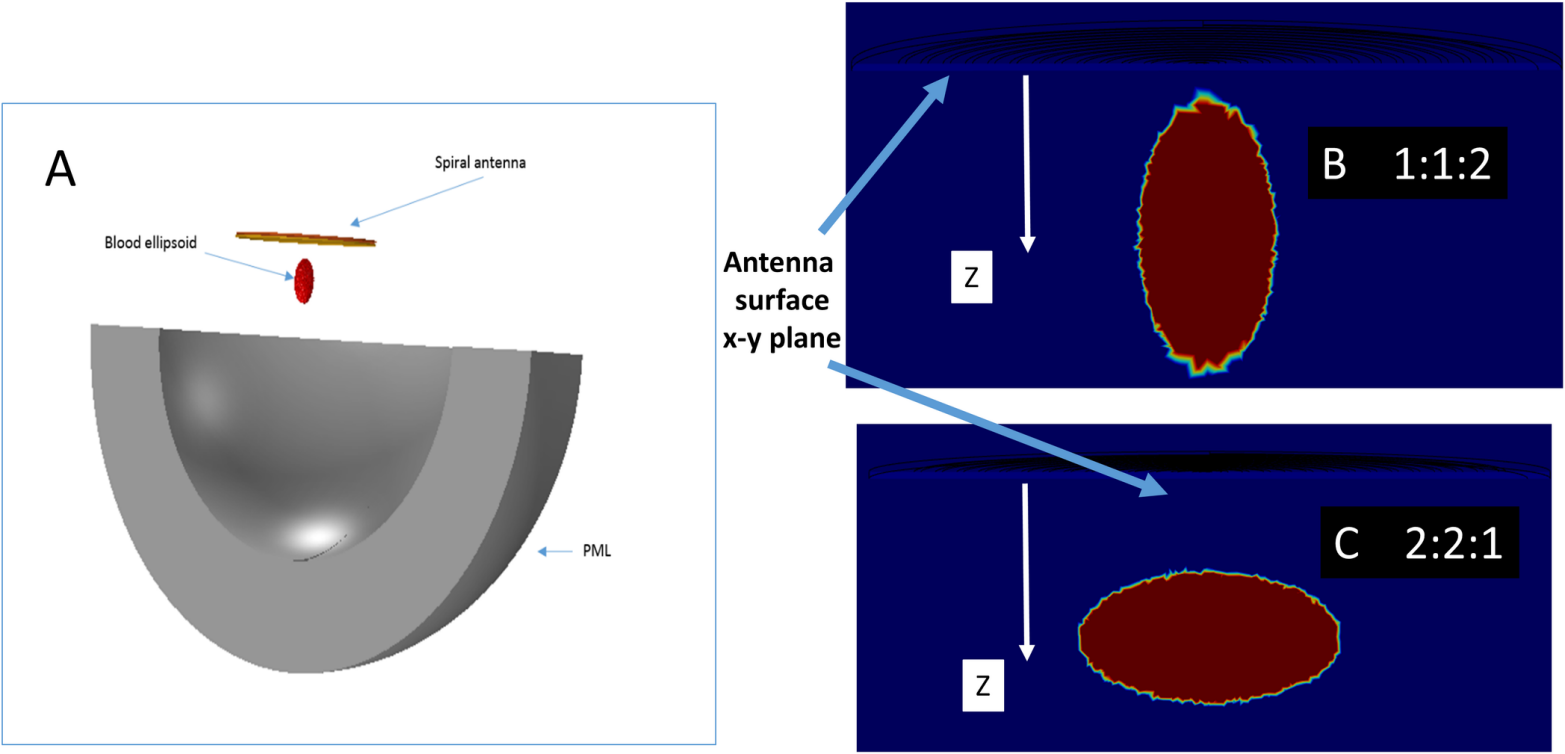
The model used to simulate the ACBH in air. (A) The complete model, (B) and (C) The two configurations of the ellipsoid shaped ACBH relative to the outer surface of the antenna facing it.

The analysis of ACBH in free air was done to study the effect of different radar relevant parameters on an ACBH, isolated from the effect of the head. Fig 4A, shows the model used to study the effect of an ACBH in air on the antennae reading. The parameters of interest are the volume of the ACBH, the cross section exposed to the incident electromagnetic waves coming from the antenna, and the wavelength. The ACBH was modeled with the dielectric properties of blood[52]. To examine the effect of the cross section we employed an ACBH shaped as an ellipsoid. We placed the ACBH in two configuration relative to the antenna outer surface (x-y plane). In Fig 4B the long axis of the ACBH is in the z direction, normal to the x-y antenna surface (at the ratio of 1:1:2 between the x, y, z respectively) and in Fig 4C, the short axis of the ACBH is in the z direction, normal to the x-y antenna surface (where the ratio between the x, y, z axes is 2:2:1, respectively). The volume was varied from 0 ml to 50 ml. The antenna used is a spiral antenna with a radius of 75mm, as described in the materials and methods section. The model was surrounded by PML (Perfect match layer). The ACBH center of gravity is placed 5 cm below the antenna surface along the centerline axis, in air. The analysis was performed for frequencies of 100 MHz, 500 MHz and 1 GHz.

**Fig 5:**
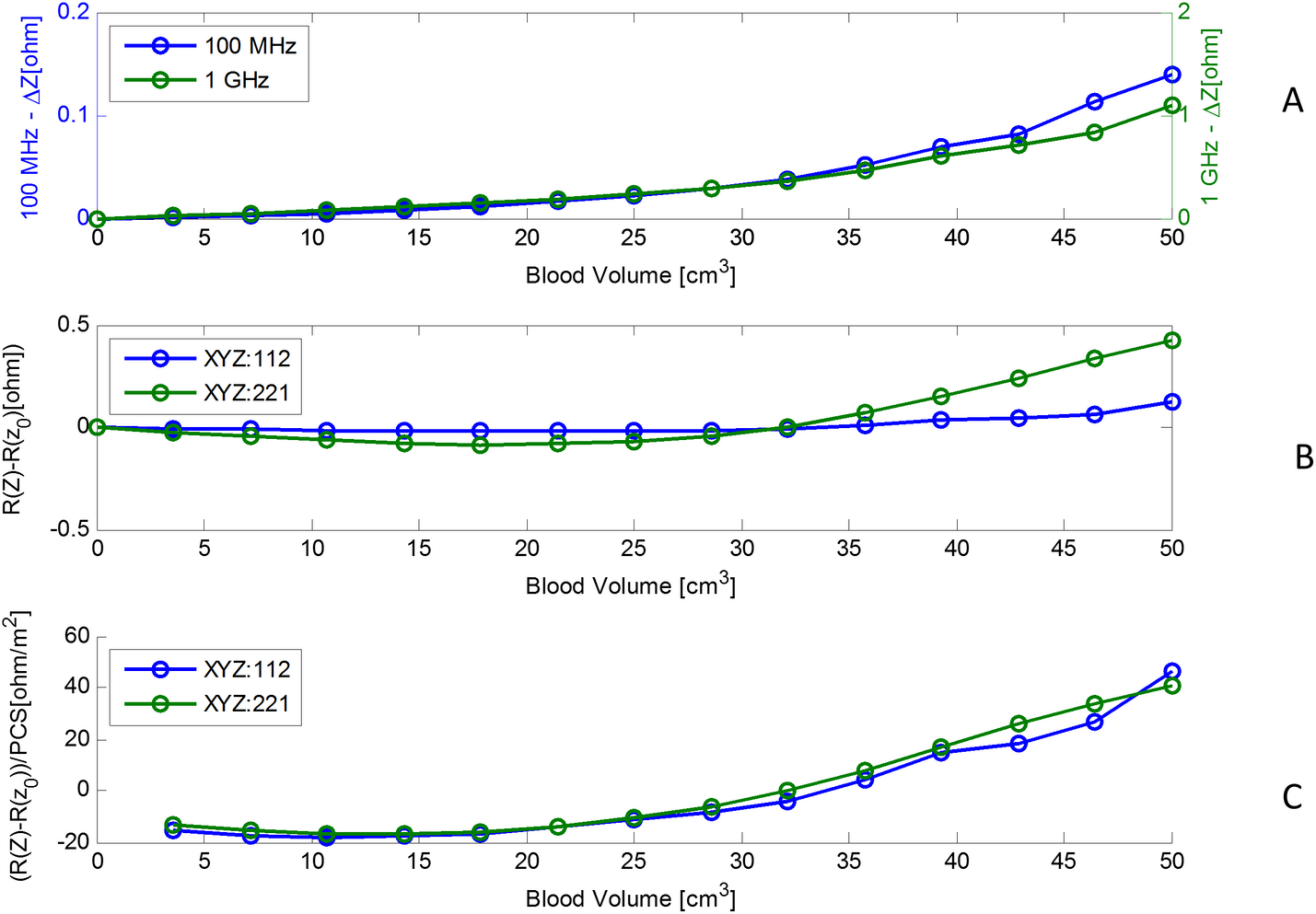
Results obtained from the simulation of the ACBH in air. The abscissa is the volume of the ACBH. (A) The change in absolute input impedance as a function of volume for two radar frequencies 100 MHz and 1GHz. (B) The real part of the change in input impedance as a function of the ACBH volume for the two ellipsoid orientations, as in 4B and 4C. (C) The real part of the change in input impedance, normalized with respect to the geometrical cross section area facing the antenna monitoring the blood, as a function of the blood volume for the two ellipsoid orientations, as in 4B and 4C.

Results from the analysis are displayed in Fig 5. The abscissa is the volume of the ACBH. In this study we used the volume as the variable parameter, because volume of blood is used in clinical practice. A more precise measure would be the geometrical cross section facing the antenna, but this has little practical clinical applicability. Fig 5A, shows the effect of the blood volume on the absolute antenna's impedance change, as calculated from Eq 3. In this case the orientation of the ellipsoid is (x,y,z– 1:1:2 as in Fig 4B). Fig 5A compares results for a frequency of 100 MHz with those for a frequency of 1 GHz. It is obvious that the effect of volume change is not linear and that the changes in absolute impedance at a probing frequency of 1 GHz, is an order of magnitude larger than the changes in absolute impedance at a probing frequency of 100 MHz. This leads to an important observation and suggests that a higher frequency can provide a greater measurement sensitivity. The concept of the radar cross section, *σ*, facilitates an understanding of the results and provides insight in the use of an antenna for monitoring ACBH. The RCS dependence on frequency (Eq 5) results from two effects: the ACBH reflection coefficient dependency on frequency and, the ratio between the wavelength and the dimension of the object. The reflection coefficient of blood at lower frequency is higher than the reflection coefficient at higher frequency [55]. However, for clinically relevant dimensions of the ACBH and radiofrequency electromagnetic waves, the RSC is in the Mie and Rayleigh regions. The RSC effect becomes dominant, leading to the results in Fig 5A.

Fig 5B and 5C, are presented to provide further insight into the effect of the RCS. The figures display results for two different configurations of the blood as shown in Fig 4B and 4C. In Fig 5B, the ordinate is the change in the real component of the impedance and the abscissa is the blood volume. We display here only the real part of the change in impedance because, the radar equation is concerned only with the real part of impedance. The two curves show results for the same volume of blood, but with different orientations of the ellipsoid, i.e. a different cross section is presented to the radar. It is interesting to notice that in one of the curves, the impedance actually decreases with an increase in the volume of the ACBH. This is important for clinical situations, where the ACBH radius/wavelength ratio places the RCS in the Mie region. In this region the RCS is not monotonic with respect to ACBH radius/wave length ratio, and can oscillate. Therefore, an increase in ACBH radius can result, for certain wavelengths, in a decrease in impedance, yielding misleading clinical information; i.e. if only the response to a single wavelength is taken as an indication of growth or decrease in the ACBH size. An important conclusion, with clinical relevance, is, that, when the growth of an ACBH is monitored with measurements produced by electromagnetic waves, it will be necessary to consider the radar cross sections in the analysis. One possible way is to analyze the response from several frequencies to avoid misleading information due to measurements in the Mie region, and to develop special frequency weighted monitoring algorithms as in [24]. This is consistent with our experimental observations when we developed the multifrequency phase shift spectroscopy technology [19, 20]. Fig 5B, also shows that, for the same volume of ACBH, there are different changes in impedance as a function of the orientation of the ellipsoid. This is the effect of the cross section presented to the radar. A larger cross section, will produce greater changes in impedance.

The effect of the Projected Cross Section (PCS), i.e. the surface that faces the electromagnetic waves incident from the radar antenna on the radar measurements, is demonstrated in Fig 5C. Here, the changes in impedance were normalized to the radar facing PCS. It is seen that when the change in impedance is normalized to the PCS, the two different curves in Fig 5B, become superimposed.

Fig 5B and 5C illustrate an important conclusion, that the projected cross section facing the radar is the dominant parameter affecting the radar reading and not the volume of the ACBH. For clinical practice, this means that it is important to place the radar antenna relative to the ABCH in an optimal orientation relative to the PCS, to maximize detection sensitivity.

### Simulated ACBH in a computer model of a real head

**Fig 6:**
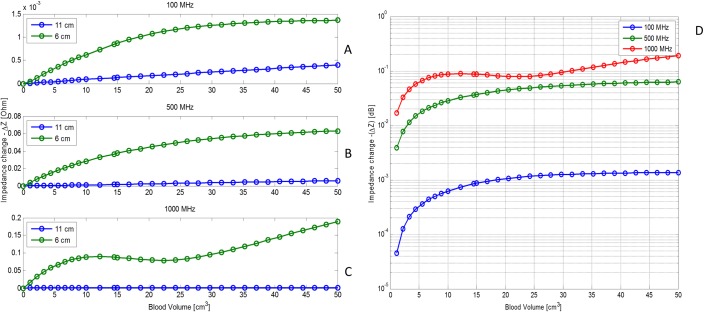
The change in absolute input impedance at two different distances from the antenna. The ordinate is the change in absolute input impedance. The abscissa is ABCH volume. (A) 100 MHz, (B) 500 MHz, (C)1 GHz, D) all three frequencies (at a distance of 11 cm) on a logarithmic scale.

**Fig 7:**
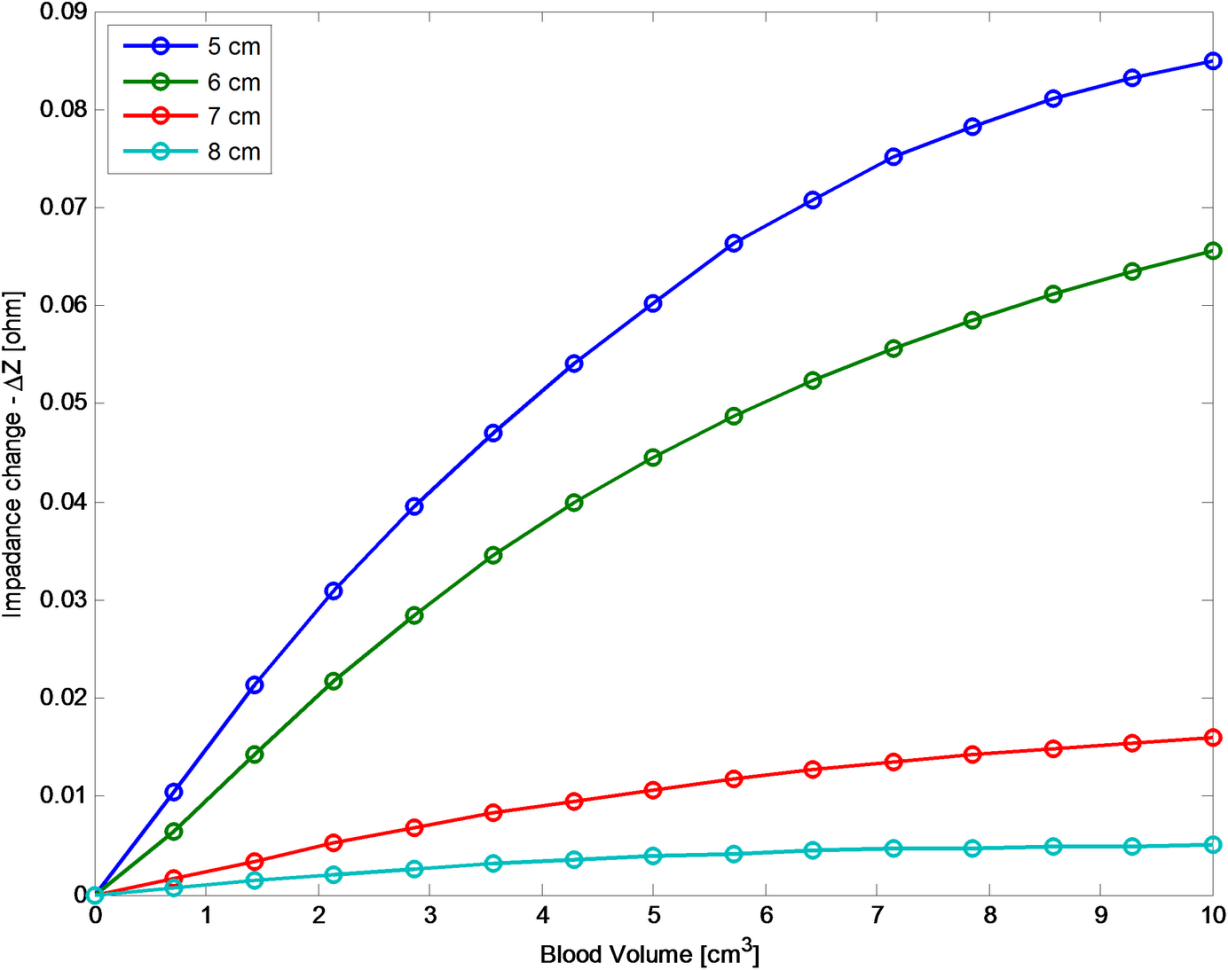
The change in absolute impedance input for different distances between the antenna and the ACBH. The ordinate is the change in absolute input impedance, when using a frequency of 1 GHz. The abscissa is ACBH volume.

**Fig 8:**
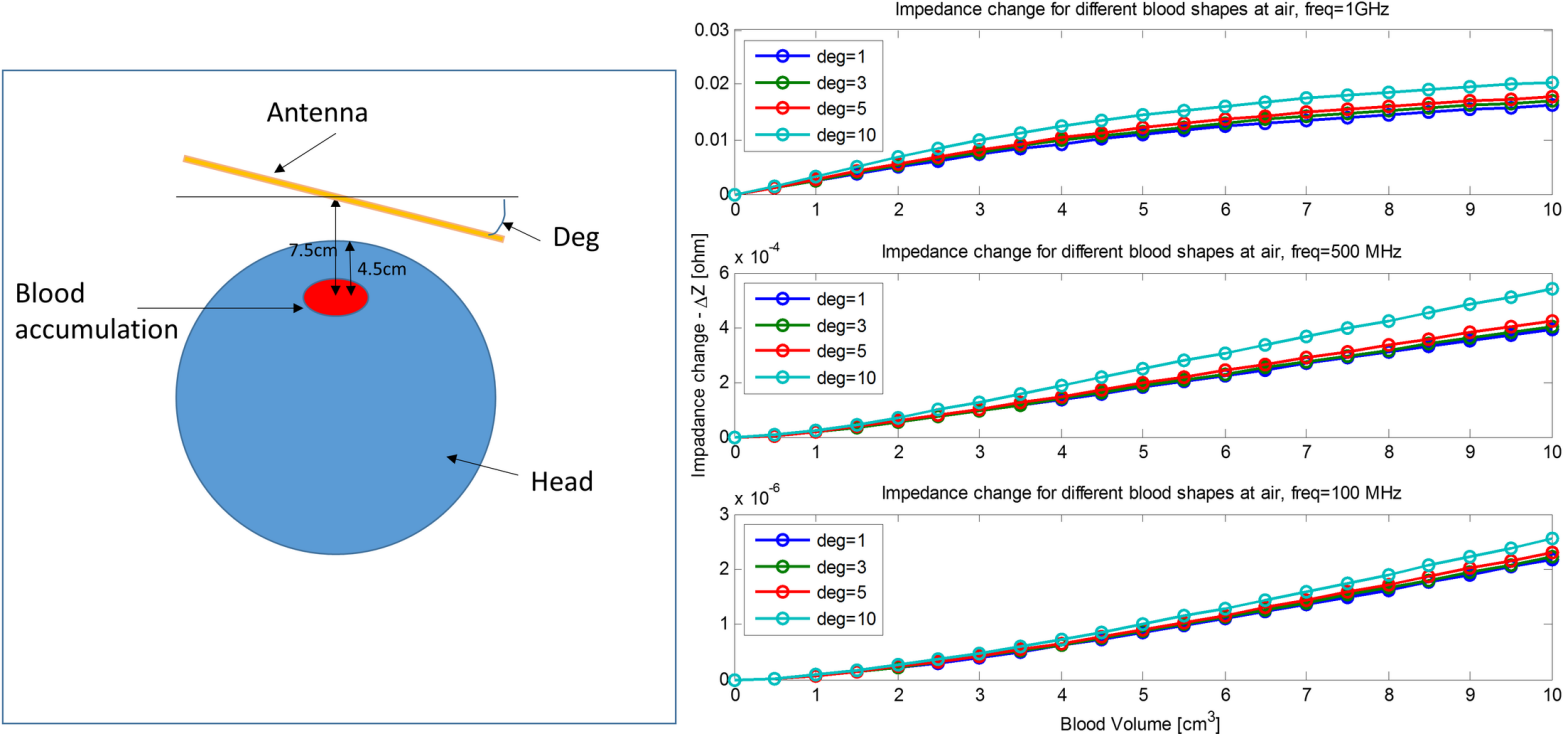
The change in absolute impedance input for different angles between the antenna and XY plan. The ordinate is the change in absolute input impedance (for 100, 500, 1000 MHz). The abscissa is ACBH volume.

In this part of the study, we use mathematical modeling to examine the effects of different parameters on monitoring ACBH in a real head, with radar. The head image used and the mathematical model are described in Fig 2. The results in Figs 6–8 and S1–S3, were all obtained for the brain model shown in Fig 2. The ACBH was simulated by an ellipsoid with axes ratio of 2:2:1 (Fig 4B). The placement of the ACBH ellipsoid is shown in Fig 2, from different view angles. The antenna was placed normal to the long axis of the ellipsoid, as in Fig 4B. The center of gravity of the ellipsoid was set at two distances from the center of the antenna, 6 cm and 11 cm. The volume of blood ranged from 0 to 50 ml. The frequencies examined where, 100 MHz, 500 MHz and 1 GHz.

In Fig 6, we examine a situation in which the antenna is at a fixed location and the ACBH is at different distances from the antenna. The purpose of the study in Fig 6, was to understand the effect of the location of the ACBH in the brain on the radar reading. Panels 6A, 6B and 6C, show the change in absolute impedance (Eq 3) for frequencies of 100 MHz, 500 MHz and 1GHz, respectively and two different distances of the center of gravity of the ellipsoid (Fig 4B) from the center of the radar surface, 6 cm and 11 cm. Comparing the panels shows that the higher the frequency, the larger the value of the absolute change in impedance and, obviously, the greater the resolution. The difference between the reading at 100 MHz and 1 GHz is two orders of magnitude and between 500 MHz and 1 GHz is a factor of four. This aspect is more evident in Fig 6D, where the impedance change with volume, is plotted on a logarithmic scale. One important conclusion from Fig 6A, 6B and 6C is that higher frequencies will produce a larger signal and therefore greater resolution.

Fig 6A, 6B and 6C, also show that the magnitude of the reading decreases with an increase in the distance between the ACBH and the radar surface. The relative decrease is larger at higher frequencies. Therefore, while the higher frequencies produce higher signal, they are more sensitive to variations in the depth of the ACBH.

While higher frequencies produce a higher resolution signal, Fig 6C and 6D, illustrate the problems with higher frequencies. The RCS of typical clinical ACBH volumes may land in the Mie region or in the Rayleigh region, as a function of the radar frequency used. Therefore, increase in volume may not always result in an increase in the absolute impedance. Fig 6C and 6D, show that there are realistic situations in which the absolute impedance is actually decreasing, while the volume of the ACBH is increasing. This is a serious drawback of the radar method, when a single frequency is used for monitoring. A possible solution is to use multiple frequencies in the monitoring. For instance, while the resolution of the 500 MHz reading in Fig 6B, is lower than that of the 1 GHz reading in Fig 6C, it displays only increases in impedance with volume. It is possible that the use of radar to evaluate ACBH will require developing multifrequency algorithms, in which different frequencies may be given different weights, which is a topic that requires further research. Another possibility is to develop frequency measurements based classifiers, as done by us with multifrequency transmission measurements in the head in [22]. In the future, we will also examine different other combinations of the radar data, in addition to the one used in this study, to seek a way to optimize the information from the radar reading. For example, measurements that give greater weight to the phase shift as in [19, 20, 22].

In Fig 7, we examine a situation in which the ACBH is at a fixed location in the brain and the antenna moves, relative to the skull. The purpose of this analysis is to examine the effect of possible head or antenna movements during the monitoring of the ACBH growth. The figure is for a configuration similar to that shown in Fig 4C, i.e. an ellipsoid in which the shorter axis is in the z direction, normal to the plane of the antenna surface. The center of gravity of the ACBH is 4.5 cm from the outer surface of the skull. The figure lists values of the distance between the surface of the antenna and the center of gravity of the ellipsoid. For example, when the distance is 5 cm, the antenna surface is 0.5 cm from the surface of the skull and when the distance is 8 cm, the antenna is 3.5 cm from the skull. The frequency is 1GHz for these cases (similar results were obtained for all the frequencies). An important outcome is that the value of the impedance is sensitive to distance between the antenna surface and the ACBH. However reasonable readings can be obtained even from a distance of 3.5 cm between the antenna surface and the skull. This should substantially simplify the clinical use of the radar technology. The primary purpose of the radar measurement technology, whose feasibility is examined in this study, is to monitor changes in the blood volume. We anticipate that, if a change is observed, the physician will be alerted and conventional imaging will be used to find the clinical details of the change. From Fig 7, it appears that as long as the distance of the antenna to the head is maintained constant, it will detect the sought after information on the occurrence of a change in ACBH size, independent on the exact value of the distance between the antenna and the blood lesion. An important consideration in designing a radar based ACBH monitoring device, is to maintain a constant distance from the head. It appears that maintaining a constant distance from the ACBH is probably more important than the actual distance. From the analysis in this section, it is evident that a precise and permanent positioning of the antenna relative to the ACBH, is important. This is possible by designing a fixture that rigidly attached to the head, as in our previous studies [23, 24].

In the previous studies, we have assumed that the plane of the antenna is normal to the line connecting between the center of the ACBH and the center of the antenna. Here we evaluate the situations in which the angle is not normal. The schematic is depicted in Fig 8 on the left panel and the results are shown in Fig 8 on the right panel. The measurements are affected by the angle. Until 5 degrees the changes are small and they increase at 10 degrees. Since the goal of this technology is to monitor change in size, variations in angle will not affect the function of the device. In fact, it seems that the resolution may increase with a change in angle, as one part of the antenna is brought closer to the examined blood lesion.

This paper deals with a mathematical analysis of the feasibility of monitoring ACBH with radar. In previous figures we have examined the effect of various design parameters on monitoring ACBH with radar, with particular emphasize on the relations between frequency, volume of blood and location. In the following figures we will examine the effects of numerical errors in the analysis and the effects of signal to noise.

To obtain an estimate of the possible errors from modeling S1 Fig examines the effect of adding an error to the electrical properties of blood used in the analysis. The errors were added to the data in the literature [52], in the form of white Gaussian noise with magnitudes of 1%, 3% and 5%. The electrical properties in each modeled voxel at a location (i,j,k) were changed according to the formula:

Property(i,j,k)=(1±Random(Gaussianwhitenoisepercentage))×(Propertyfromliterature) (6)

In this way, each voxel experienced a different random error. The model was a ACBH sphere with a volume ranging from 0 to 4.25 cm^3^. The center of gravity of the sphere was located at a distance of 7 cm from the center of the antenna surface. The antenna surface was 0.5 cm above the skull. The results were obtained for a 1 GHz antenna. The figure shows the relation between the measured change in impedance and blood volume for different percentage errors in electrical properties estimate. It is evident that a random error as large as 5% imposed on each voxel does not affect the overall pattern of change in impedance/ ACBH relation. The main application of the technology we evaluate here is to determine the feasibility of monitoring ACBH in time, with radar, rather than evaluating the exact volume of the ACBH. S1 Fig shows that even an error as large as 5% in the mathematical model will not affect the conclusions from the analysis in this paper–in regards to evaluating the feasibility of using radar measurements to monitor ACBH.

An important parameter in electrical circuits design is the effect of the signal to noise ratio (SNR), defined as:

$${SNR}_{dB}=20∙{log}_{10}\left(\frac{V_{signal}}{V_{noise}}\right)$$ (7)

There, are various means to reduce the SNR in an antenna, but this is not the focus of this study. Here we will examine the effect of SNR on the ability to monitor the growth of an ACBH with the radar technology. The ACBH is an ellipsoid with axes ratio of 2:2:1, as shown in Fig 4C, whose center of gravity is located 7 cm below the center of the antenna and the distance between the antenna surface and the skull surface is 0.5 cm. We examined the change in absolute impedance, when white Gaussian SNR of 40 dB or 50 dB [56] was added to the electromagnetic wave signal value entering the antenna. S2 Fig displays the calculated impedance when 40 dB or 50 dB SNR was added to the data for radar frequencies of 100 MHz, 500 MHz and 1 GHz. The fuzzy data points come from different numerical experiments in which a Gaussian signal to noise error was introduced. S3 Fig compendium, is another way to display the data in S2 Fig by plotting on the abscissa the difference between the calculated impedance value from an input with a signal to noise error and on the ordinate the number of data points that have expressed this order of magnitude of the error. Obviously, it resembles a Gaussian plot, with a peak at the origin. The observation that the effect of noise produces a Gaussian distribution error around the signal without noise suggests that multiple measurements at the same frequency will reduce the effect of noise. In fact, in our study in [23], we have used for this purpose 128 repeats at each frequency. S2 and S3 Figs, show that the effect of the error due to SNR, is lower for higher frequencies. This is an advantage of using higher frequencies for monitoring ACBH.

### Simulation of ACBH growth from various clinical data in the literature

**Fig 9:**
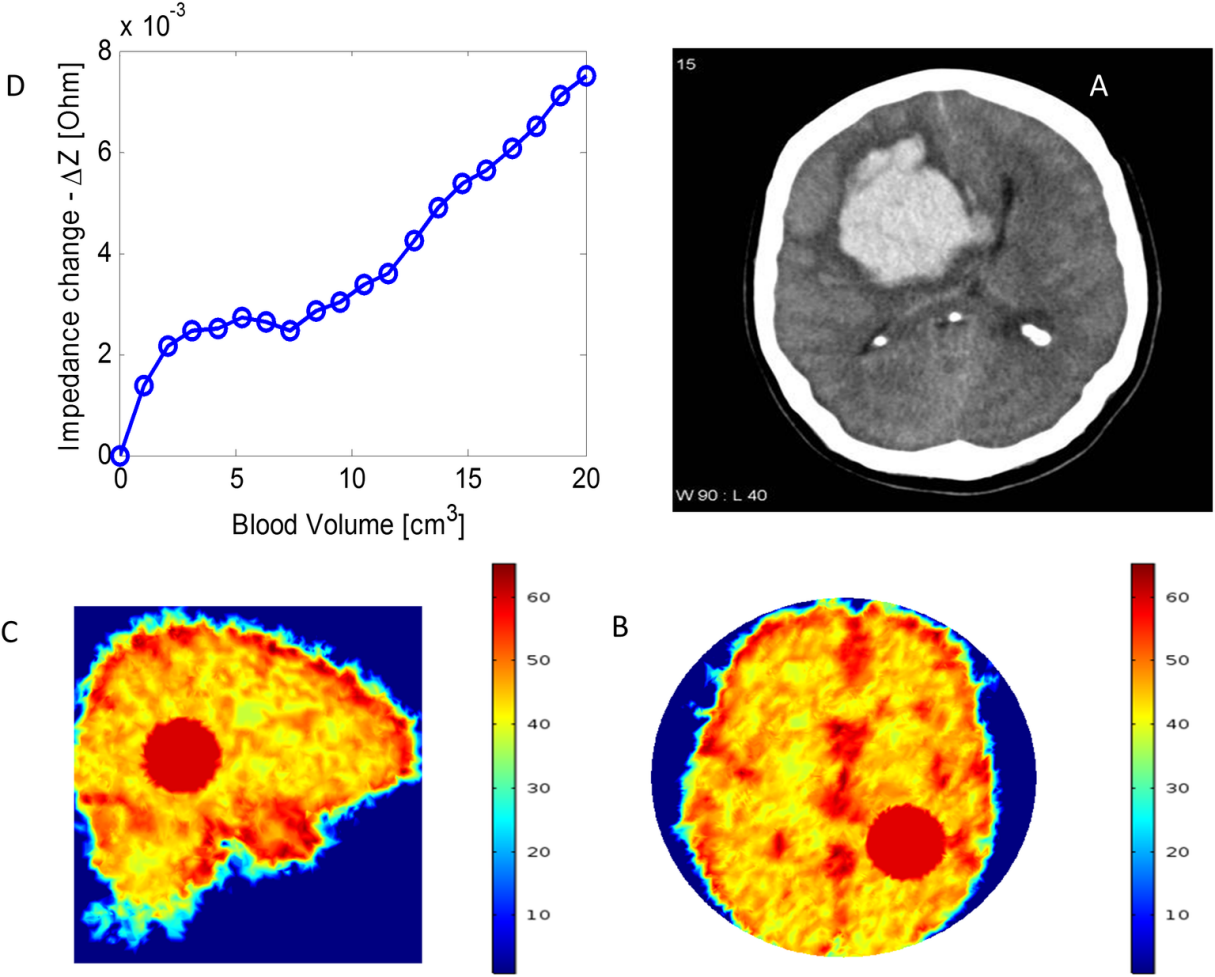
A simulation analyzing the detection of blood volume change in a subarachnoid hemorrhage with radar. (A) Image of a lobar hemorrhage from a case study by Dr. Frank Gaillard Radiopaedia.org [57]. (B), (C) The location of the ACBH on the permeability map for the case in panel A. (D) Calculated change in absolute input impedance as a function of hemorrhage volume.

**Fig 10:**
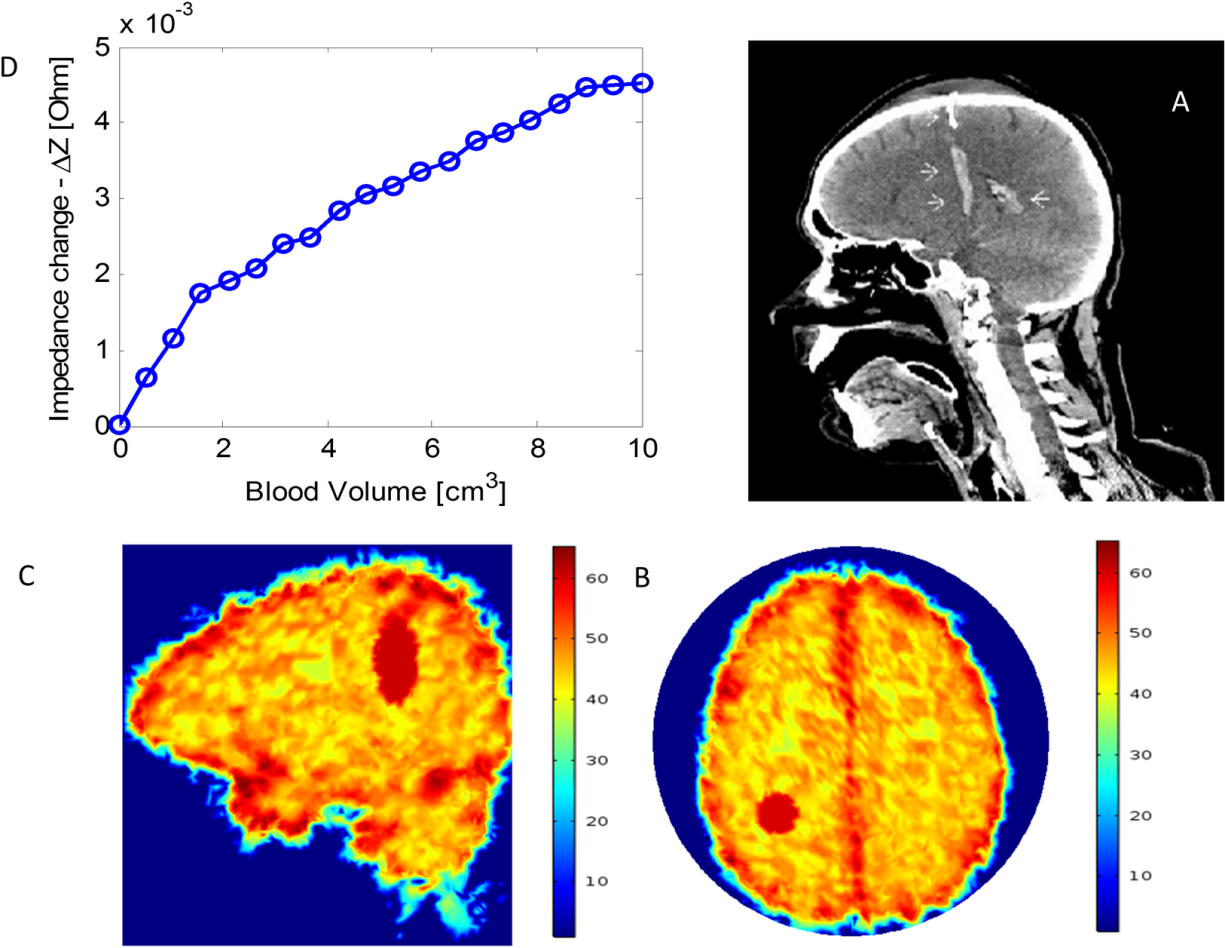
A simulation analyzing the detection of blood volume change in an intraparenchymal hemorrhage with radar. (A) The image of an intraparenchymal hemorrhage is taken from a case of spade injury by Dr. Nafisa Shakir Batta Radiopaedia.org. [56] (B), (C) The location of the ACBH on the permeability map for the case in panel A. (D) Calculated change in the absolute input impedance as a function of hemorrhage volume.

To generate a better understanding of the clinical potential of the radar technology to monitor growth of ACBH, we have gathered images of various types of ACBH from the Internet, and simulated monitoring the growth of the ACBH for these cases. These studies are shown in Figs 9 and 10. The figures show the actual CT image, the model of the ACBH and the calculated change in impedance for a radar measuring at 1 GHz.

Fig 9 is for the case of a subarachnoid hemorrhage. The clinical data, shown in panel A is taken from [57] (case 3, lobar hemorrhage). In our simulation, the hemorrhage was modeled as a sphere, located as shown in the figure (Panels B and C). The distance from the outer surface of the antenna to the skull is 0.5 cm. The location of the center of the ACBH relative to the center of the antenna facing the skull is (x = -2cm, y = -3cm, z = -10cm). The change in absolute impedance with volume is given in panel D. It can be observed that the 1 GHz radar technology could have monitored the growth of the hemorrhage, even though it is deep in the brain. However, the reading of the change in impedance is not linear with the change in volume, and, in fact are regions in which there is the dip in absolute change in impedance. As discussed earlier, this is the effect of the change in volume and the consequent radius-wavelength ratio being in the Mie region. It further emphasizes the observation that algorithms must be developed to handle these complications. A possible solution worth exploring is the use of multifrequency radar, an area of research we plan to pursue in the future.

Fig 10, is for a case of intraparenchymal hemorrhage [56]. In this simulation the antenna is assumed to be 0.5 cm from the skull. The location of the center of the ACBH relative to the center of the antenna facing the skull is (x = -3cm, y = -4cm, z = -8cm). The hemorrhage is simulated as an ellipsoid with axes ratios of 4:1:1 and where the major axis is normal to the surface of the antennae. The radar frequency is 1 GHz. An interesting observation is that for this shape of hemorrhage the impedance in Fig 10D, grows with the growth of the lesion, unlike in Fig 9D. This emphasizes the fact that the ratio between wavelength and lesion size is important, because different types of results may be obtained from different ratios. It further emphasizes the need for using multifrequency, or at least understanding the significance of the ratio between wavelength and lesions size.

## Conclusion

This is a theoretical study that has examined the feasibility and attributes of radar monitoring of the growth of a hematoma or a hemorrhage in the brain, non-invasively and from a distance, when the location of the hematoma or hemorrhage are known from conventional medical imaging. This may have value as a low cost and safe alternative to multiple conventional medical imaging follow-up of bleeding in the brain. We have developed a radar model to simulate the measurement. The numerical results applied to 3-D models of real brains, demonstrate the feasibility of using radar to monitor growth in blood volume. An important design parameter is the frequency used for monitoring the ACBH. Data obtained for frequencies of 100 MHz, 500 MHz and 1 GHz show that the resolution improves with an increase in frequency, in the range studied. However, it appears that when typical clinical dimensions of hematoma and hemorrhage are used, the variable ratio of blood volume radius and radar wavelength can bring the measurements into the Mie and Rayleigh regions of the radar cross section. In these regions the measurements can oscillate and produce misleading results. In these regions there is a oscillatory change in signal with blood volume size. For some frequencies there is an increase in signal with an increase in volume while in others there is a decrease. This is an aspect of our findings that requires further investigation. Special algorithms must be developed, when the wavelength is at the length scale of the blood volume, possible of a type that employs classifier technology, multifrequency algorithms or ultra-wide band (UWB) measurement. This is important research for the future. The results suggest that a wide-band multifrequency radar is required for practical clinical application of this radar monitoring technology.

## Supporting information

S1 Fig The change in absolute impedance input for Gaussian noise type error in the electrical parameters. The ordinate is the change in absolute input impedance for Gaussian noise type error in the assumed values of electrical parameters employing 1 GHz. The abscissa is ABCH volume.
(TIF)

S2 Fig The change in absolute input impedance for different ratio of signal to noise (SNR). The ordinate is the change in absolute input impedance for different ratio of signal to noise (SNR) at the input to the antenna, at frequencies of 100 MHz, 500 MHz and 1 GHz. Green line is the change in absolute impedance without noise, blue data points include a SNR error of 40 dB and red data points involve a SNR error of 50 dB.
(TIF)

S3 Fig The difference between the absolute change in impedance with SNR input and without. (TIF)
