# Supplementary figures and images for: Radar based technology for non-contact monitoring of accumulation of blood in the head: A numerical study
Source: PLoS One. 2017 Oct 12;12(10):e0186381. doi: 10.1371/journal.pone.0186381 (PMC5638502; doi:10.1371/journal.pone.0186381)

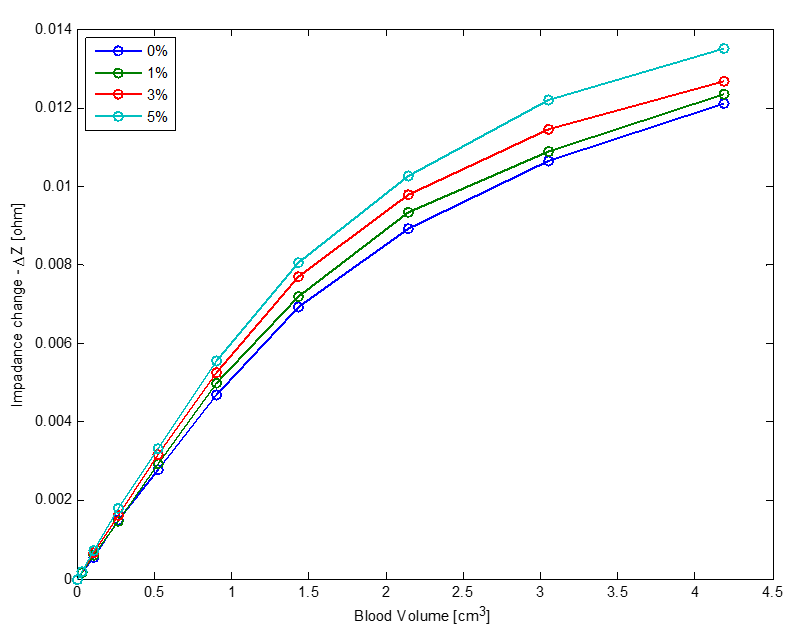

Supplement: S1 Fig — The ordinate is the change in absolute input impedance for Gaussian noise type error in the assumed values of electrical parameters employing 1 GHz. The abscissa is ABCH volume. (TIF) [file pone.0186381.s001.tif]

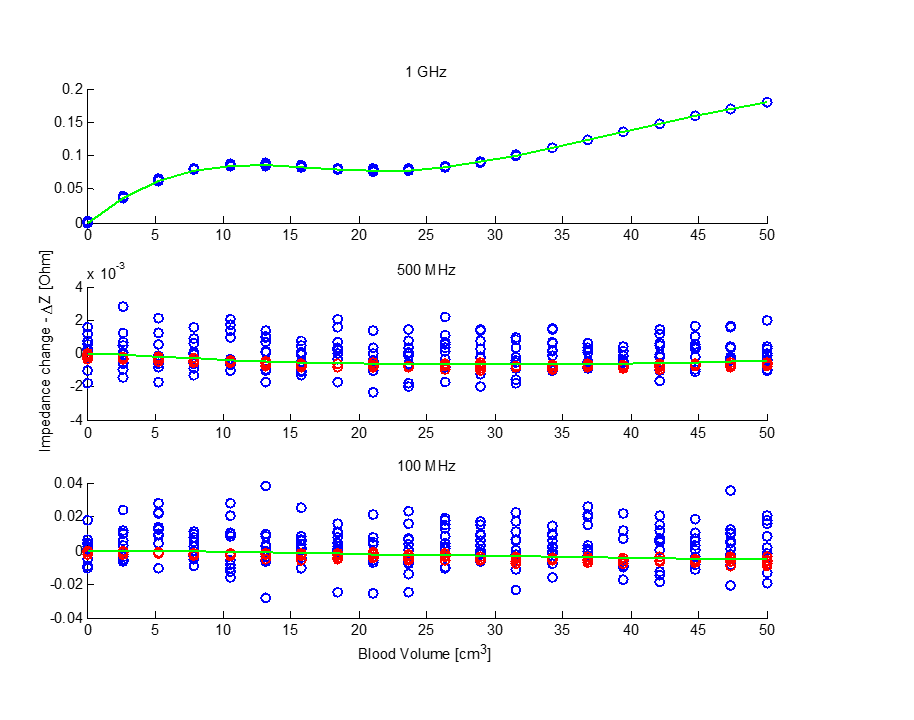

Supplement: S2 Fig — The ordinate is the change in absolute input impedance for different ratio of signal to noise (SNR) at the input to the antenna, at frequencies of 100 MHz, 500 MHz and 1 GHz. Green line is the change in absolute impedance without noise, blue data points include a SNR error of 40 dB and red data points involve a SNR error of 50 dB. (TIF) [file pone.0186381.s002.tif]

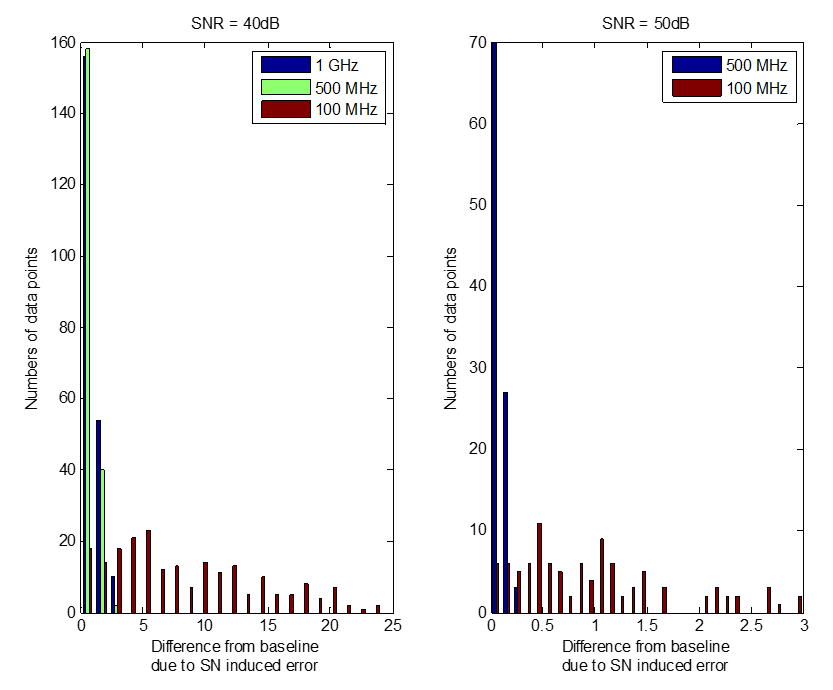

Supplement: S3 Fig — (TIF) [file pone.0186381.s003.tif]
